# Optimizing *de novo* transcriptome assembly from short-read RNA-Seq data: a comparative study

**DOI:** 10.1186/1471-2105-12-S14-S2

**Published:** 2011-12-14

**Authors:** Qiong-Yi Zhao, Yi Wang, Yi-Meng Kong, Da Luo, Xuan Li, Pei Hao

**Affiliations:** 1 Key Laboratory of Synthetic Biology, Institute of Plant Physiology and Ecology, Shanghai Institutes for Biological Sciences, Chinese Academy of Sciences, Shanghai 200032, China; 2 Institute of Massive Computing, Software Engineering Institute, East China Normal University, 3663 North Zhongshan Road, Shanghai, 200062, China; 3 State Key Laboratory of Biocontrol, Sun Yat Sen University, Guangzhou, 510275, China; 4 Shanghai Center for Bioinformation Technology, 100 Qinzhou Road, Shanghai, 200235, China

## Abstract

**Background:**

With the fast advances in nextgen sequencing technology, high-throughput RNA sequencing has emerged as a powerful and cost-effective way for transcriptome study. *De novo* assembly of transcripts provides an important solution to transcriptome analysis for organisms with no reference genome. However, there lacked understanding on how the different variables affected assembly outcomes, and there was no consensus on how to approach an optimal solution by selecting software tool and suitable strategy based on the properties of RNA-Seq data.

**Results:**

To reveal the performance of different programs for transcriptome assembly, this work analyzed some important factors, including *k*-mer values, genome complexity, coverage depth, directional reads, *etc*. Seven program conditions, four single *k*-mer assemblers (SK: SOAPdenovo, ABySS, Oases and Trinity) and three multiple *k*-mer methods (MK: SOAPdenovo-MK, trans-ABySS and Oases-MK) were tested. While small and large *k*-mer values performed better for reconstructing lowly and highly expressed transcripts, respectively, MK strategy worked well for almost all ranges of expression quintiles. Among SK tools, Trinity performed well across various conditions but took the longest running time. Oases consumed the most memory whereas SOAPdenovo required the shortest runtime but worked poorly to reconstruct full-length CDS. ABySS showed some good balance between resource usage and quality of assemblies.

**Conclusions:**

Our work compared the performance of publicly available transcriptome assemblers, and analyzed important factors affecting *de novo* assembly. Some practical guidelines for transcript reconstruction from short-read RNA-Seq data were proposed. *De novo* assembly of *C. sinensis* transcriptome was greatly improved using some optimized methods.

## Introduction

With the fast advances in nextgen sequencing technology in recent years, massively parallel cDNA sequencing (RNA-Seq) has emerged as a powerful and cost-effective way for transcriptome study. RNA-Seq has been widely applied to both well-studied model organisms and non-model organisms, to provide information on transcript profile of organisms, and to give important insights into biological processes [1-5]. For organisms with known reference genomes, researchers usually take advantage of mapping-first strategy to analyze transcriptome data. However, mapping-first strategy is not suitable when reference sequence is not available or incomplete. Thus, for organisms with un-sequenced genome or cancer cells with widespread chimeric RNAs [6,7], *de novo* assembly is essential to provide a workable solution for transcriptome analysis.

In theory, *de novo* assembly of short sequence reads into transcripts allows researchers to reconstruct the sequences of full transcriptome, identify and catalog all expressed genes, separate isoforms, and capture the expression levels of transcripts. However, in reality *de novo* transcriptome assembly faced some unique challenges. Assemblers must be tuned to handle conditions that were not present for genome assembly. Among those conditions, transcripts are expressed at both low and high levels, spanning a difference of ten thousands folds. On top of that, sequence biases from nextgen sequencing technology can further skew the expression of transcripts. Expression of gene isoforms due to alternative splicing, and expression of genes with overlapped regions would grossly compound the difficulty in *de novo* transcriptome assembly.

Until recently, a few attempts were made to handle the difficult tasks of assembling transcriptome from short-read RNA-Seq data. Most of them were modified from the breakthrough technology for genome assembly using short sequence reads. SOAPdenovo [8], ABySS [9], and Velvet-Oases (hereafter referred as Oases) [10] were reported to be successfully applied to transcriptome assembly of various organisms [3,9,11-13]. More recently, Grabherr *et al*. [14] released Trinity, a program specially developed for *de novo* transcriptome assembly from short-read RNA-Seq data, which was shown to be efficient and sensitive in recovering full-length transcripts and isoforms in yeast, mouse and whitefly. Trinity constructed *de Bruijn* graph from large amounts of short-read sequences, then used an enumeration algorithm to score all possible paths and branches, and retained those plausible ones as transcripts/isoforms. Trinity was specially programmed to recover paths supported by actual reads and remove ambiguous/erroneous edges, thus ensured correct transcript reconstruction.

On the other hand, a different strategy, which employed multiple *k*-mer (MK) values in building *de Bruijn* graph in order to handle both highly and lowly expressed transcripts, was proposed by Robertson *et al*. [11], and by Surget-Groba and Montoya-Burgos [15]. While all *de Bruijn* graph-based assemblers were programmed using a single optimal *k*-mer length based on that whole-genome shotgun sequencing libraries provided a uniform representation of genomic sequences, non-normalized mRNA libraries can present a wide expression range of transcripts in addition to transcript isoforms due to alternative splicing events. Thus, it was likely that MK presented a strategy advantageous over single *k*-mer (SK) for optimized assembly of transcripts at different abundance.

With the challenges facing *de novo* transcriptome assembly and emerging solutions from several research groups, there has not been a consensus on what variables to consider for choosing a suitable tool, how to approach an optimal solution based on available information on data, and even more importantly how to design an efficient transcriptome study with maximizing reward by taking advantage of available assembly tools. We designed this study to evaluate the performance of publicly available assemblers for short-reads RNA-Seq data: SOAPdenovo, ABySS, trans-ABySS, Oases and Trinity. Oases was specially designed for transcriptome assembly, extended from its corresponding Velvet version developed for genome assembly. SOAPdenovo and ABySS were originally developed for genome assembly and also applied in transcriptome assembly. In this study, we compared SK and MK strategies, and examined how various coverage depths affected assembly outcomes. In order to understand how genome complexity influences transcriptome assembly, we used two model organisms: *D. melanogaster* and *S. pombe*, which differed in genomic properties. By running repeat tests on identical machine, we gained the information on assemblers’ resources requirement, memory usage, and runtime. In addition, we applied the different methods to reconstruct the transcripts for *C. sinensis*, an important economic cultivar used to produce a good variety of tea products. We were able to significantly improve on previously assembled transcriptome result by reconstructing more full-length and high-quality transcripts with more RNA-Seq reads incorporated.

## Materials and methods

### RNA-Seq data sets

RNA-Seq data sets used in this study were all publicly available, and could be retrieved from NCBI SRA database. They included a standard (non-strand specific) Illumina data set from fruit fly, *D. melanogaster*, a strand-specific data set from fission yeast, *S. pombe*, and a standard data set from tea plant, *C. sinensis*.

The *Drosophila melanogaster* data (Dme-data) were 76bp paired-end (76PE) Illumina reads. Their accession codes are: SRR023199, SRR023502, SRR023504, SRR023538, SRR023539, SRR023540, SRR023600, SRR023602, SRR023604, SRR027109, SRR027110, SRR027114 and SRR035403. Dme-data were obtained from mixture of *D. melanogaster* embryonic samples from 0 to 24 hours after egg laying [1]. The *Schizosaccharomyces pombe* data (Spo-data) were strand-specific 68PE Illumina reads. Its accession code is SRP005611. Spo-data came from four biological conditions, including late stationary phase, heat shock, mid-log growth and growth after all glucose has been consumed [14]. The *Camellia sinensis* data (Csi-data) were 75PE Illumina reads. Its accession code is SRX020193. Csi-data included samples from seven different tissues of *C. sinensis*: tender shoots, young leaves, mature leaves, stems, young roots, flower buds and immature seeds [3].

### Preprocessing RNA-Seq data

Dme-data were preprocessed before used for *de novo* assembly: reads that did not contain at least 41 Q20 bases among the first 51 cycles were removed. Q20 base refers to the base with Q-value≥20, which is defined as an error probability ≤ 1%. Low quality (<Q20) 3’ end of reads were then trimmed off by custom PERL script. After preprocessing, we obtained totally 13.08 G bases (Gb) quality filtered short reads data (~ 106.8 Million read pairs). We randomly sub-sampled read pairs in *D. melanogaster* quality filtered data set to generate 0.5 Gb (~ 4.1 M read pairs), 1 Gb (~ 8.3 M read pairs), 3 Gb (~ 25.0 M read pairs), 5 Gb (~ 41.7 M read pairs) and 7 Gb (~ 58.3 M read pairs) subsets. Spo-data and Csi-data were used without preprocessing step, thus to keep the same data sets used in previous studies [3,14]. It has been reported that 50 M paired-end *S. pombe* reads (~ 6.8 Gb) were almost saturated for *de novo* assembly [14]. Thus, we randomly subsampled read pairs in Spo-data to generate 50 M subset (~ 6.8 Gb) as well as three smaller subsets, 0.5 Gb (~ 3.7 M read pairs), 1 Gb (~ 7.4 M read pairs) and 3 Gb (~ 22.1 M read pairs) for purposes of analysis. For Csi-data, all of the short reads (2.32 Gb, ~ 15.46 M read pairs) were used for the analysis.

### *De novo* assembly

Transcriptome short reads were *de novo* assembled using SOAPdenovo (release 1.05)[8], ABySS (version 1.2.7)[9], Velvet (version 1.1.04)[16] followed by Oases (version 0.1.21)[10] or Trinity (release 20110519) [14]. We assembled each data set using similar assembly parameters (*k*-mer value = 25, CPU = 20), thus trying to keep the same condition to compare their performance. The Command-line parameters used with SOAPdenovo were “-K 25 –p 20 -R -d -F”; ABySS: abyss-pe k=25 n=10 j=20 name=xx in='fq1 fq2'; Velvet(multithreaded)-Oases: “-cov_cutoff 2”; Trinity: --CPU 20 --bfly_opts "--edge-thr=0.05 --compatible_path_extension" for *D. melanogaster* and *C. sinensis* datasets; --CPU 20 --SS_lib_type RF --jaccard_clip --bfly_opts "--edge-thr=0.05 --compatible_path_extension" for *S. pombe* dataset (strand specific), also tested without --jaccard_clip option for Spo-6.8g data set. Trans-ABySS was run by using a set of *k*-mer values including 19, 25, 31, 37, 43 and 49, and then merged assembled results by the first step of trans-ABySS analysis pipeline. MK strategy was also applied to SOAPdenovo and Oases using the same *k*-mer set and merged by the first step of trans-ABySS analysis pipeline. All the assemblies were performed on a server with 48 cores and 512 G of memory. The operating system is Ubuntu 10.04 LTS. After assembly, only transcripts with no less than 100 bases were used for the downstream analysis.

### Removal of redundancy

For MK strategy, merging all transcripts from different *k*-mer assemblies will introduce redundancy. What’s more, for some assemblers, occasionally, constructed transcripts will also show redundancy (shorter transcript was entirely covered by longer one with 100% identity). For this scenario, CD-HIT-EST was used to remove the shorter redundant transcripts when they were entirely covered by other transcripts with 100% identity. This set of transcripts was then aligned to CDS sequences and genomes for the assessment. Since some isoforms of reconstructed transcripts were different only for small variations, such as SNPs, small insertions or deletions, this may introduce bias for the basic assembly statistics. CD-HIT-EST was used to remove the shorter redundant transcripts when they were 100% covered by other transcripts with more than 99% identity. The non-redundant transcripts were then used to count the basic assembly statistics for each method.

### Mapping reads to transcripts

To get assembly statistics for the number of reads that could be mapped back to transcripts (RMBT) , we used bowtie (version 0.12.7) [17] to map back all input short reads to the reconstructed transcripts, with parameters “-q --phred33-quals --fr -1 fq1 -2 fq2 -v 3”.

### Mapping reconstructed transcripts to reference

Genome sequence and gene annotations for *S. pombe* (version 09052011) were downloaded from the ftp site of Sanger institute (ftp://ftp.sanger.ac.uk/pub2/yeast/pombe/). Genome data for *D. melanogaster* was downloaded from download page of UCSC genome browser (http://hgdownload.cse.ucsc.edu). Existing gene models were downloaded from UCSC Table Browser, and only the Ref genes were used to evaluate the performance of each assembler. For the protein coding sequences, a custom PERL script was applied to remove the redundancy for those exactly identical sequences: the original 22680 protein coding transcripts of *D. melanogaster* and 5174 transcripts of *S. pombe* were reduced to 18558 and 5150 non-identical coding transcripts, respectively. BLAT[18] with default parameters was applied to map the reconstructed transcripts from each assembler to non-identical reference coding sequences and reference genomes. Four groups of hits were classified for the evaluation of the capability for CDS reconstruction: 1) Covered the entire reference coding sequence, having no mismatch, insertion or deletion (100%); 2, 3, 4) At least 95%/80%/50% sequence identity covering the entire reference coding sequence, respectively. To assess the accuracy of reconstructed transcripts, we aligned reconstructed transcripts to the reference genome using BLAT and then the number of equal or more than 95% or 50% of reconstructed transcripts that could be aligned back to its corresponding genome was used for the assessment. Transcript with less than 50% of its length could be mapped back to the genome was defined as unmapped-transcript. Shared and unique transcripts parsed from pairwise alignments were aligned to the reference genome. Transcript with at least 95% of its length could be aligned to corresponding genomic locus was considered for the assessment.

### Expression quintiles

Short reads used for assembly were aligned to the CDS sequences by Tophat (v1.2.0) [19], and then custom PERL scripts were applied to computing normalized gene expression level by calculating RPKM (Reads Per Kilobase of exon model per Million mapped reads) of each transcript. Only paired end mapped reads were considered in this study. Gene was defined as expressed if it’s RPKM >0, and then all expressed genes were divided into expression quintiles at 10% intervals for the evaluation.

## Results

### Study design and RNA-Seq data collections

Currently five publicly available assemblers have been reported to be used for *de novo* assembling short-read RNA-Seq data into transcripts. They are SOAPdenovo, ABySS, trans-ABySS, Oases and Trinity. Trans-ABySS was developed by ABySS team that adopted MK strategy to ABySS. Following the same approach, we applied MK strategy to SOAPdenovo and Oases (referred as SOAPdenovo-MK and Oases-MK, respectively). Trinity, on the other hand, fixed its *k*-mer value at 25 that was not changeable. It used a specially designed algorithm to recover possible transcripts/isoforms to ensure high plausibility. But at the meantime, to assemble the same dataset Trinity required runtime at least 20 folds more than the other programs used under SK condition. So we found it impractical to apply MK strategy to Trinity at current stage. Thus, our design included 7 program conditions: 4 with SK (SOAPdenovo, ABySS, Oases and Trinity) and 3 with MK (SOAPdenovo-MK, trans-ABySS and Oases-MK). All the tests were run on the same single-node machine with 512G memory and 4 AMD Opteron 6168 (12-core) processors.

In order to examine how genome with different complexity affects assembly outcomes, we selected public RNA-Seq data from two model organisms as benchmark: fruit fly (*D. melanogaster*) and fission yeast (*S. pombe*). Fruit fly has a genome size of 117 Mb, having 22680 protein coding genes and average intron length ~ 2.3kb (based on RefSeq gene sets). Fission yeast has a smaller genome of ~ 12.5 Mb [20], with 5174 protein coding genes, and average intron length ~ 81bp. Besides both organisms have excellent genome reference available, their distinct genome properties helped elucidate how simple (fission yeast) or more complex (fruit fly) genomes influenced transcriptome assembly. Tea plant, *C. sinensis*, has a large genome (~ 4G) yet to be resolved. We hoped to significantly improve on its existing transcriptome assembly, so to demonstrate the usefulness of optimizing strategy and guidelines for *de novo* transcriptome assembly.

### Comparison of transcript assembly under different program conditions

In order to compare the performance of each assembler, we put in test two sets of benchmark data that displayed different data properties. In addition, we varied the amount of initial inputs from the two sets of data to evaluate the effect of coverage depths on the assembly outcomes (details in *Materials and Methods*). The outcomes are summarized in Additional file 1 and 2.

When measured in the number of assembled transcripts, total bases of transcripts, mean length, N50, percentage of low quality transcripts, number of long-transcripts (≥1kb), and number of reads that could be mapped back to transcripts (RMBT), we observed significant improvement on the outcomes when MK strategy was applied to each program. For all paired tests: SOAPdenovo *vs*. SOAPdenovo-MK, ABySS *vs*. Trans-ABySS, and Oases *vs*. Oases-MK, there were at least 50% increases in the number of assembled transcripts, total bases of transcripts, and number of long-transcripts comparing MK to SK (Additional file 1 and 2).

With increasing coverage depth, each assembler generally produced a larger number of transcripts and more total bases, but the mean transcript length and N50, after an initial increase, peaked at a certain threshold and started to decrease. The percentage of RMBT had a pattern reversely correlated to increasing coverage depth for all program conditions except for Trinity.

Overall, Oases-MK assembled the most transcripts and long-transcripts, whereas trans-ABySS/ABySS produced the longest mean transcript length and the largest N50. While Trinity preformed the best in the percentage of RMBT, SOAPdenovo was the worst in the category. The percentage of RMBT is an important benchmark for evaluating the performance of each method. An optimal program should use as many reads as possible to reconstruct high-quality transcripts. Trinity reached almost 90% with the *D. melanogaster* data, which may be attributed to its greedy *k*-mer-based approach at the Inchworm step. Oases-MK came in second for this measure. Given the number of low quality transcripts, performance of SOAPdenovo was not satisfactory.

### Resources usage by different assemblers

**Figure 1:**
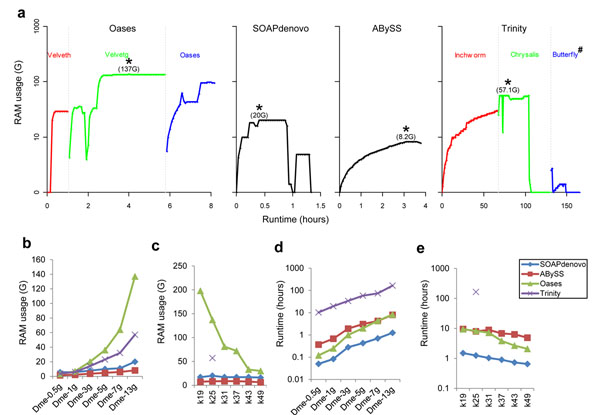
**Runtime and RAM usage performance for each assembler.** Runtime and RAM usage for each assembler: Oases, SOAPdenovo, ABySS and Trinity. (a) Real-time monitored runtime and RAM usage of each method using Dme-13g data set. The maximum RAM usage was marked as asterisk for each assembler, and three stages of Oases and Trinity were shown by different colors (red: Velveth and Inchworm; green: Velvetg and Chrysalis; blue: Oases and Butterfly). RAM usage (b) and runtime (d) of each method using different amounts of inputs with *k*-mer value of 25. RAM usage (c) and runtime (e) of each method using Dme-13g data set with different *k*-mer value. #Alternatively, jobs from Butterfly module could be distributed in clusters using a job array, which could greatly reduce the running time for this step.

The demand for resources to carry out *de novo* assembly is an important factor to consider when choosing a software tool. While it was proved to be critical in assembly of large genome, resources usage for assembling transcripts bears some equal importance for practical reason. We monitored and recorded the runtime and memory usage for four SK assemblers running on testing data sets on the same computer. We found the runtime and memory usage were two essential factors that limit the use of a program. The measured data of runtime and memory occupancy for each assembler tested with SK method are illustrated in Figure 1.

The four SK assemblers displayed distinct memory usage patterns through their processing steps. Among them, Oases consumed the largest maximum memory (at Velvetg step), whereas memory usage by ABySS was the smallest (Figure 1a). It was assumed that larger data set would consume more memory. This was generally true with all four assemblers as the memory usage displayed a good correlation with the size of testing data (Figure 1b), though Oases was the most sensitive, and ABySS the least sensitive in response to increasing data size. The *k*-mer values also had great impact on both memory usage and runtime. Memory usage displayed reverse correlation with *k*-mer values for Oases but remained constant for SOAPdenovo and ABySS (Figure 1c, Trinity remains unknown as its *k*-mer value was not changeable). While Trinity required the longest runtime and SOAPdenovo the least for the same testing dataset, the time costs for all four tools, as expected, were approximately proportionate to the size of testing data set (Figure 1d). Runtimes for ABySS, Oases, and SOAPdenovo were reversely correlated with the *k*-mer values (Figure 1e), but the impact was not as dramatic as that of *k*-mer values on memory usage.

These results indicated that assembly using Oases with small *k*-mer value requires large memory and may exceed the memory space of a typical computing sever nowadays, and processing of a large data set by Trinity can exceed reasonable execution time and hence becomes impractical. Thus these factors warrant careful consideration when one chooses a tool for analysis as well as setting parameters associated with the tool.

### Validating assembled transcripts by mapping to reference genome

**Figure 2:**
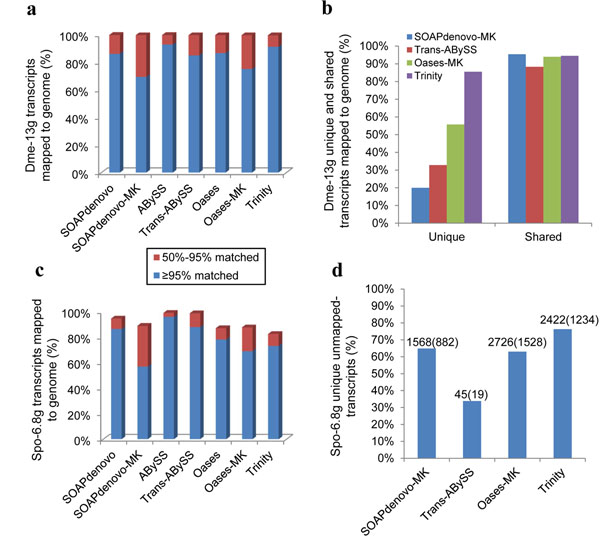
**Number of transcripts that could be aligned to the genome**. Shown are the percentages of transcripts that could be successfully aligned to its corresponding genome with Dme-13g (a) and Spo-6.8g (c) data sets. (b) Percentage of unique and shared transcripts that could be successfully aligned to the genome using Dme-13g data set by each of SOAPdenovo-MK, trans-ABySS, Oases-MK and Trinity. (d) The percentage of unique unmapped-transcripts produced from each of assembly methods using Spo-6.8g data set. Numbers above the histogram are the number of unique unmapped-transcripts (left) and number of unique unmapped-transcripts that had BLASTX top hits (E≤10^-10^) to Uniprot database (right, within the brackets).

To validate assembled transcripts, we mapped each transcript to its reference genome as described in *Materials and Methods*: *Map reconstructed transcripts to reference*. Transcripts assembled from *D. melanogaster* data sets using different methods showed a high percentage in alignment to its reference genome. Less than 0.5% of assembled transcripts failed to align (Figure 2a, shown using Dme-13g data set), and similar results were found using smaller sampling data from *D. melanogaster* data sets (data not shown). Pairwise alignment using BLAT was performed for transcripts from SOAPdenovo-MK, trans-ABySS, Oases-MK and Trinity. Shared (defined as at least 95% sequence identical between two transcripts from different methods) and unique (if the transcript is not shared, then it was unique) transcripts were then aligned to genome separately. While the shared transcripts were generally validated by mapping to genome at a high percentage, the unique ones were mapped to reference genome at various levels with Trinity being the best and SOAPdenovo the worst (Figure 2b).

For *S. pombe* data set, Trinity, Oases and Oases-MK showed worse performance than for *D. melanogaster* data set, with more than 10% transcripts failing to be aligned to reference (Figure 2c). Unique transcripts accounted for more than 60% of all unmapped-transcripts (Figure 2d) except for trans-ABySS (33.83%). Except for trans-ABySS (19/45), the rests had over 50% of unique unmapped-transcripts with BLASTX hits (E≤10^-10^) to Uniprot database [21] (Figure 2d), representing some *bona fide* gene transcripts. We further tested whether low quality sequence in *S. pombe* data set contributed to the high percentage of unmapped-transcripts. After trimming low quality nucleotides (<Q20) from 3’-end before re-assembly, Trinity had a 6~7% increase in matched transcripts (data not shown), confirming that sequence errors in *S. pombe* data set were at least part of the reason for the higher level of unmapped-transcripts.

### Evaluating gene coverage and integrity of assembled transcripts

**Figure 3:**
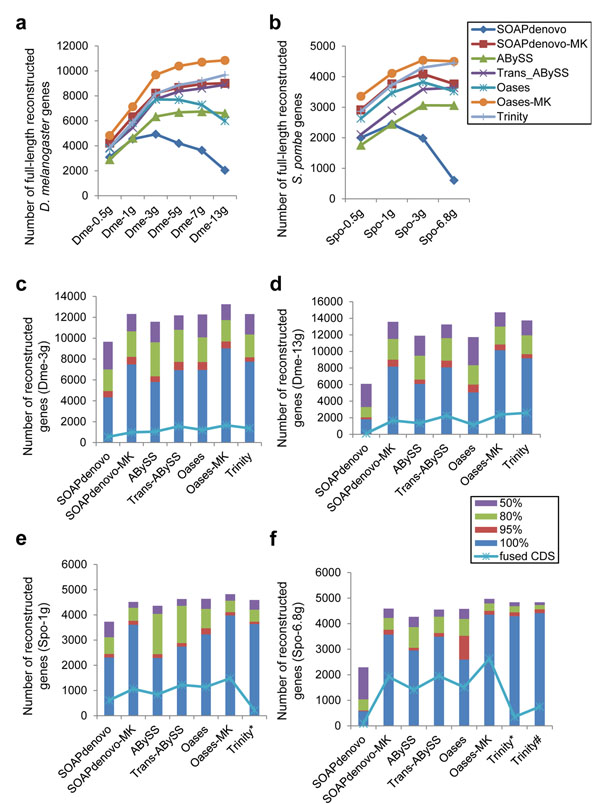
**Number of reconstructed protein coding genes**. Number of full-length protein coding genes reconstructed by each method using inputs with different depth of coverage: *D. melanogaster* data sets (a), *S. pombe* data sets (b). Number of reconstructed genes were shown using Dme-3g (c), Dme-13g (d), Spo-1g (e) and Spo-6.8g (f) data sets, which included full-length reconstructed genes with 100% (blue) and at least 95% identity (reddish brown); partial-length reconstructed genes: 80% (green) and 50% (purple). Trinity assembly with strand specific option “--SS_lib_type RF” was marked as asterisk. The assessment of Trinity without “--jaccard_clip” option was shown as “Trinity^#^” using Spo-6.8g data set (f).

The gene coverage and transcript integrity are important performance benchmarks for transcriptome assembly. We evaluated gene coverage and transcript integrity with *D. melanogaster* and *S. pombe* data sets by matching reconstructed transcripts to CDS and examining the numbers of covered full-length genes. The full-length transcripts reconstructed by different program conditions displayed some similar patterns: the numbers of full-length transcript initially went up with increasing sequence reads; in cases of SOAPdenovo-MK, ABySS, trans-ABySS, Oases-MK and Trinity their numbers leveled off at certain data levels, whereas for SOAPdenovo and Oases their numbers started to drop (Figure 3a, b). The turning points appeared to be related to the complexity of the genome. The turning point was around 3G for fruit fly, and between 1-3G for fission yeast.

For *D. melanogaster*, there is totally 55.46Mb of unique transcripts from RefSeq or 53.80Mb from Ensemble gene sets. Assuming 80% of the genes expressed, the 3Gb-sequence reads, where the turning point was observed, amounts to ~75× average coverage on total expressed genes. For *S. pombe*, the turning point equals to approximately 100× average coverage. These numbers are important reference in design of future *de novo* transcriptome study, in which some estimate and careful testing are recommended to find the optimized parameters for a given organism. Full-length, partial-length, and fused CDS were illustrated for transcripts reconstructed from *D. melanogaster* (Figure 3c, d) and *S. pombe* (Figure 3e, f) data sets. At the curve-turning point or the full-data point, MK methods appeared to build more full-length CDS comparing to SK with same assemblers, whereas partial-length CDS remained almost unchanged. On the other hand, there was an increase in the numbers of fused CDS being associated with the MK methods.

It’s worth noting that the number of fused genes was low for *S. pombe* transcripts reconstructed by Trinity, which took use of strand-specific information for assembly (Figure 3e, f). This was not observed with *D. melanogaster* transcripts, where no strand-specific information was available. In addition, Trinity had a “--jaccard_clip” option that was recommended for gene dense genome with lots of transcripts overlapping on the same strand. For *S. pombe* transcripts, the option significantly reduced the number of fused genes (Figure 3f, personal communication with Brian J. Haas).

In comparison of different program conditions, Oases-MK appeared to cover the most in number of genes as well as the most in number of full-length genes. While comparable in total number of assembled transcripts, SOAPdenovo-MK and trans-ABySS were lagging in the number of reconstructed full-length genes (Figure 3c, d, e, f). For SK methods, Oases’s performance was satisfactory at small data set, but lagged behind with increased inputs. Again, SOAPdenovo was the worst performer for this measurement, especially with large inputs data at high coverage depth.

### Evaluating sensitivity of assemblers to genes expressed at different levels

**Figure 4:**
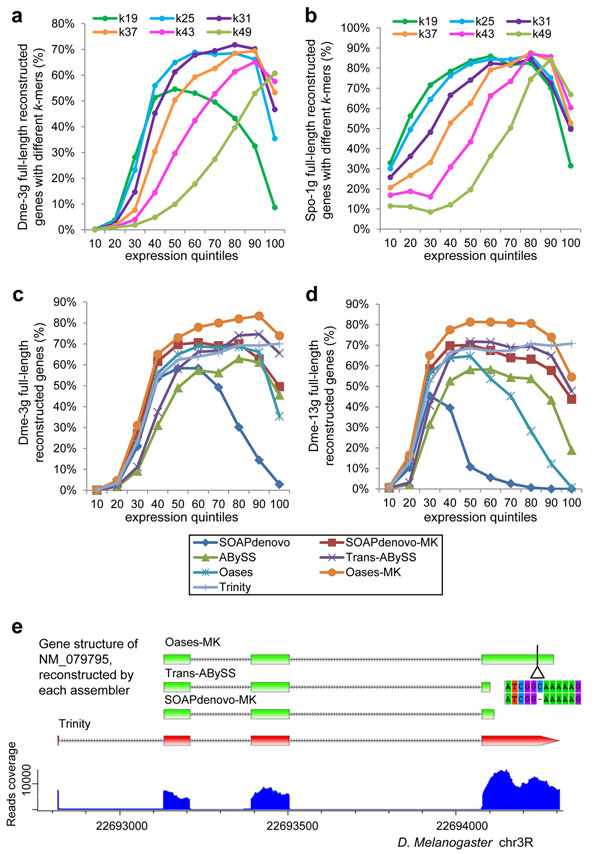
**Full-length genes reconstructed by each method at different expression quintiles**. Shown are the percentages of reconstructed full-length genes (Y axis) at different expression quintiles (X axis, 10% increment) by Oases with different *k*-mer values using Dme-3g (a) and Spo-1g (b) or by each assembler using Dme-3g (c) and Dme-13g (d) data sets. (e) An example is shown as an assembled transcript in *D. melanogaster* by different assembly methods. NM_079795 is one of the highly expressed genes at highest expression quintile, which could be completely reconstructed by Trinity (red), but failed by other methods. Only incomplete transcripts (green) were reconstructed and both ends of coding region were lost. Incomplete transcript with 1 bp deletion assembled by Oases-MK is shown below its gene model. Reads coverage is shown at the bottom.

The sensitivity of program condition to gene expression level was examined by counting the full-length transcripts of various expression levels. As shown in Figure 4a and 4b, using varying *k*-mer values Oases captured transcripts in a different range of expression quintiles. The small *k*-mer value, i.e. *k*=19, worked better for transcripts at low quintiles, whereas a large *k*-mer value, i.e. *k*=49 only worked in a high quintile range. On the other hand, the MK methods took advantage of these properties from different *k*-mer values, and can cover transcripts in a broad expression range (Figure 4c, d).

Comparing the different program conditions, our data showed that all had a poor performance at 10%~30% lowest quintiles (Figure 4c, d). Surprisingly, Trinity reconstructed a steady number of CDS at above 30% quintiles. The others, SOAPdenovo, Oases, and ABySS when using SK strategy did not perform well for either the lowly or the highly expressed genes. However, when employing MK strategy, the performance of SOAPdenovo, Oases, and ABySS was greatly improved, especially on the high quintile levels (Figure 4c, d). We observed that highly expressed genes were often assembled into incomplete transcripts. As shown in Figure 4e, NM_079795 represents one of the highly expressed genes in *D. melanogaster*. While Trinity correctly reconstructed the entire transcript of NM_079795, various short forms were generated by other program conditions.

### *De novo* assembly of *C. sinensis* transcriptome by different assemblers

The tea plant, *Camellia sinensis*, is one of the most important economic cultivar that is used to produce a good variety of tea products. It has an estimated genome size of about 4.0Gb [22]. With its large genome size and no genome draft being available, the transcriptome analysis provided a good option to study the gene composition, genetic polymorphism, and metabolic basis of this important economic plant. However, there were some great challenges researchers faced. They included unknown number of genes in *C. sinensis*, potentially very large genetic diversity of the studied population, and unclear evolution history, *etc*.

We performed *de novo* assembly analysis to the published RNA-Seq data set from *C. sinensis *[3], which consisted of 15.46 million pairs of 75bp Illumina sequence reads. To calibrate the system and make our results comparable to the original published work (used SOAPdenovo), we first tested different *k*-mer values with SOAPdenovo, and found *k* =25 produced similar results with N50 and mean transcript length comparable to the recently published results (Additional file 3: columns “Published data” and “SOAPdenovo”). Then we performed *de novo* assembly using different program conditions on the *C. sinensis* RNA-Seq data (basic statistics are shown in Additional file 3). Overall, the MK methods (SOAPdenovo-MK, trans-ABySS and Oases-MK) produced much larger numbers of transcripts (≥100bp) with more total bases than the original published assembly data and SOAPdenovo results we obtained. SOAPdenovo-MK, trans-ABySS and Oases-MK also produced superior results in mean length, N50 and numbers of long-transcripts (≥500bp and ≥1kb) than the original published results. Within SK methods, Trinity generated significantly better results than the original published assembly data and SOAPdenovo results in almost all categories except mean length and N50. The better assemblies by MK methods and Trinity were translated into larger numbers of coding proteins. We observed significant increases in BLASTX hits to Uniprot database [21] and in the numbers of unique Uniprot proteins identified (Additional file 3). These additional genes would certainly help reveal the complete metabolic pathways in *C. sinensis* and identify the missing genes in natural molecule synthesis important to tea flavor and quality. One good example is Cinnamate 4-hydroxylase (C4H, EC1.14.13.11), which is an important enzyme that converts cinnamate to p-coumarate in flavonoid biosynthesis pathway. In the original paper [3], it was indicated that there was no cinnamate 4-hydroxylase in *C. sinensis*. However, in our assembly results from either Oases-MK or Trinity, while performing BLASTX against the KEGG database [23], we were able to identify multiple C4H gene transcripts (Additional file 4 and 5) that filled into the gap in flavonoid biosynthesis pathway.

## Discussion and conclusions

*De novo* assembly of transcriptome from short-read RNA-Seq data presented some unique challenges to bioinformaticians. This study was designed to evaluate the performance of five publicly available assemblers that were previously used to assemble short-reads transcriptome data: SOAPdenovo, ABySS, trans-ABySS, Oases, and Trinity. In order to reveal the important factors to consider for choosing an optimal strategy and software tool, we set up variable testing conditions: single *k*-mer *vs*. multiple *k*-mer, simple genome *vs*. complex genome, low coverage depth *vs*. high coverage depth, non-directional reads *vs*. directional reads, *etc*. We measured results in terms of resources usage, transcript accuracy, integrity and completeness, and sensitivity to assemble transcripts from low to high expression levels. By analyzing and comparing the assembled results from various conditions, we were able to develop some useful guidelines that help direct future transcriptomics studies.

### Performance by different tools using SK method

Trinity had a consistently better performance in almost all the categories than the other SK tools, on the cost of longer runtime (sometimes 20~100× longer). SOAPdenovo, although using less memory and runtime, was the least satisfactory. It performed poorly for reconstructing CDS and for measurements like low quality transcripts and RMBT. Other assemblers: ABySS and Oases, had an impaired performance when reconstructing transcripts of high coverage depth. We observed that highly expressed transcripts were often incompletely assembled. However, its reason remains unclear to us and we can only speculate that sequence repeats or homologous genes may be the cause.

The size of sequencing data from Illumina platform is often very large, and therefore required substantial memory and long computing time, even for the very efficient *de Bruijn* graph-based assemblers. For large datasets, Oases required the largest memory, and Trinity took the longest runtime. ABySS and SOAPdenovo showed some good balance between memory usage and runtime.

### MK strategy enhancing performance compared to SK method

We for the first time applied MK strategy to SOAPdenovo and Oases, and systematically evaluated the performance of MK *vs*. SK on 3 assembler tools. By taking use of different *k*-mer values, the MK method was able to capture both lowly expressed transcripts with small *k*-mer value and highly expressed genes with large *k*-mer value. This strategy ensured recovering more assembled full-length transcripts at very low redundancy. The MK method appeared to work well across all spectrums of coverage depth, and with all programs tested. There can be further improvement if MK strategy is applied to Trinity. However, the application is limited to its long runtime and fixed *k*-mer value, so it is impractical to apply MK strategy to Trinity with the current version.

We observed a decrease in transcripts mapping to reference genome and increase in fusion genes by MK method when compared to SK method of the same tools. It may indicate that MK method can lead to and accumulate some incorrect assemblies or artificially fused transcripts. Given the longer and more diverse transcripts reconstructed by MK methods, the benefits clearly outweigh the pitfalls. We observed some interesting results that showed Trinity reduced the number of fused transcripts by taking use of strand-specific read information in assembly, which suggested that strand-specific sequencing was useful to tease apart overlapping transcripts on opposite strands.

The benefits of MK strategy were most demonstrated by the results from *de novo* assembly of RNA-Seq data from *C. sinensis*. The numbers of transcripts (≥100bp) and long-transcripts (≥1kb) were doubled or even tripled with MK strategy for different assemblers. There is certainly much room for improvement on reducing the artifact and redundant transcripts, which remains the main focus of future study on MK methods.

### Effects of coverage depth and genome complexity

The effect of sequence coverage depth on assembly outcome showed some interesting patterns. With the exception of SOAPdenovo and Oases, the others had generally increased number of full-length genes corresponding to increased coverage depth. Such positive correlation seems to reach plateaus at 3G data point for fruit fly. The 3G data point is also the turning point for SOAPdenovo and Oases, where the number of full-length gene assemblies started to decrease. For *S. pombe*, which has a much smaller genome compared to that of fruit fly, the turning point was between 1 and 3G. These results suggest the turning point is intrinsic to each organism, probably related to the complexity of their genome: number of genes/transcripts, average size, gene density, range of expression levels, *etc*. The genome properties of fruit fly and fission yeast were most related to their numbers of genes (22680 *vs*. 5174). The estimated number of genes is certainly important basis for designing a transcriptome experiment.

### Useful guidelines for *de novo* transcriptome assembly

It is impossible to choose an optimal tool and computation parameters for transcriptome assembly without comprehensive understanding the performance of various tools and program settings at work. By comparing the performance of these tools and assembly outcomes from variable test conditions, we recommended some basic and useful guidelines to help people choose the best tools and strategy, and to optimize program settings for transcriptome assembly work. We also summarized some shortcomings and limitations for programs and methods, hopefully for people to avoid or improve on them. In light of our results, the followings are recommended for selecting the optimal tools and conditions for *de novo* transcriptome assembly studies:

1) Generally, MK approach should be considered to achieve better assembly results.

2) Trinity is the best SK assembler for transcriptome assembly for both small and large data set across various conditions. But don’t choose Trinity if long running time is to be avoided.

3) Oases-MK and trans-ABySS produce the most diverse long transcripts. But one must avoid Oases if machine memory is limited.

4) SOAPdenovo uses smallest memory and shortest runtime. But one should avoid SOAPdenovo in general if full-length genes and complete transcriptome are desired, especially for large amounts of sequence inputs with high coverage depth.

5) Large data set can be divided into a serious of 0.5, 1, 3G subsets to test for the optimal conditions for assembly. 

6) For design a transcriptome study, usually 100× average coverage on estimated size of expressed transcripts is recommended to start with for *de novo* assembly.

## List of abbreviations used

SK: single *k*-mer; MK: multiple *k*-mer; NCBI: National Center for Biotechnology Information; SRA: Sequence Read Archive; KEGG: Kyoto Encyclopedia of Genes and Genomes; UCSC: The University of California, Santa Cruz; RPKM: Reads Per Kilobase of exon model per Million mapped reads; Dme-data: *Drosophila melanogaster* data; Spo-data: *Schizosaccharomyces pombe* data; Csi-data: *Camellia sinensis* data; RMBT: the number of reads that could be mapped back to transcripts.

## Competing interests

The authors declare that they have no competing interests.

## Authors' contributions

Q-YZ designed and performed the experiments, and drafted the manuscript. XL conceived the study, and drafted and revised the manuscript. YW and Y-MK collected data and performed analyses. DL and PH advised on experiments, data analysis, and the manuscript. All authors read and approved the final manuscript.

## Supplementary Material

Additional file 1 **Basic statistics for *de novo* assembly with *D. melanogaster* data sets.** The outcomes of transcript assemblies by each method: SOAPdenovo, SOAPdenovo-MK, ABySS, trans-ABySS, Oases, Oases-MK and Trinity. Assembled transcripts with no less than 100 bases are included. Low quality transcripts are defined as transcripts with more than 5% ambiguous nucleotides. ^#^Since scaffolding system hasn’t been built in Trinity yet, the measure of low quality transcripts for Trinity is left as “-”. Click here for file

Additional file 2 **Basic statistics for *de novo* assembly with *S. pombe* data sets.** The outcomes of transcript assemblies by each method are shown. Click here for file

Additional file 3 **Basic assembly statistics and BLASTX hits to Uniprot database using *C. sinensis* 2.3g data set.** The outcomes of transcript assemblies by each method and measurements in the previous study are shown. ^§^Some measurements are not available in the previous study, which are left as “-” in the table. Click here for file

Additional file 4 **List of C4H related transcripts assembled by Trinity and Oases-MK.** BLAST results against the KEGG database with E-value ≤ 1.0e^-5^, and only transcripts with top blastx hits to Cinnamate 4-hydroxylase (EC1.14.13.11) are shown. Click here for file

Additional file 5 **Sequences of C4H related transcripts assembled by Trinity and Oases-MK.** Fasta formatted sequences of C4H related transcripts that were listed in Additional file 4 are shown. Click here for file
